# Global scientific production on gasless laparoscopy: a bibliometric analysis

**DOI:** 10.3389/fsurg.2024.1416681

**Published:** 2024-08-09

**Authors:** Javier Pérez-Reátegui, Brad Jhefferson Arge-Gamarra, Renato Díaz-Ruiz, Akram Hernández-Vásquez

**Affiliations:** ^1^ Facultad de Ciencias de la Salud, Universidad Científica del Sur, Lima, Peru; ^2^ Hospital III Jose Cayetano Heredia, EsSalud, Piura, Peru; ^3^ Epidemiology and Health Economics Research, Universidad Científica del Sur, Lima, Peru; ^4^ Centro de Excelencia en Investigaciones Económicas y Sociales en Salud, Vicerrectorado de Investigación, Universidad San Ignacio de Loyola, Lima, Peru

**Keywords:** bibliometrics, minimally invasive surgical procedures, laparoscopy, humans, pneumoperitoneum bibliometrics, pneumoperitoneum

## Abstract

**Objectives:**

To characterize the bibliometric characteristics of the global scientific production of original research on gasless laparoscopy in the Web of Science Core Collection (WoSCC) platform.

**Materials and methods:**

A bibliometric study of original articles published up to the year 2023 was carried out. Articles were included following the selection criteria in the Rayyan web application, indexed in the Scopus database. The bibliometric analysis was performed using the Bibliometrix program in the R programming language and VOSviewer. The bibliometric characteristics evaluated were articles, journals, citations, publications, ten most mentioned articles, journals with the highest number of publications, authors and institutional affiliations; and cooccurrence of terms.

**Results:**

A total of 223 publications were included, with the highest number of articles being published in the years 1999 and 2014. The publication with the most citations was found to be a randomized trial by Galizia G in 2001 with 132 citations. We identified 846 authors involved in the production of articles on gasless laparoscopy, with Nakamura H being the most productive author with 15 articles between the years 2007 and 2020, followed by Takeda A and Imoto S, all three affiliated with “Gifu Prefectural Tajimi Hospital”. The country with the highest production was Japan with 64 publications, followed by China and Italy with 46 and 18 publications, respectively. In the top 10 journals with the highest number of publications, “Surgical Endoscopy—Ultrasound and Interventional Techniques” is in first place with 20 articles published on gasless laparoscopy; in addition, most of these are located in Q1 and Q2. Regarding the terms or keywords, it was found that the initial studies had terms related to the disadvantages of pneumoperitoneum and later focused on more specific topics of the application of gasless laparoscopy.

**Conclusions:**

Production on gasless laparoscopy has stagnated, with the topics of interest currently being its application in new, less invasive techniques. The most productive countries are found in the Asian and European continents, with little information collected in Latin America. This fact makes it necessary to increase the production of studies to promote this technique and its possible advantages.

## 1 Introduction

Lack of access to timely surgical interventions causes high morbidity and mortality in less developed countries (1). The supply of professionals per 100,000 inhabitants in these countries is 5.5, being as low as 0.7, in stark contrast to the 56.9 that can be found in more developed countries (2). On the other hand, more than 90% of the world's population, mainly living in less developed regions, lack timely access to surgery (1), resulting in delayed and often inadequate treatment (3).

Surgery is the first-line management in multiple pathologies. Likewise, minimally invasive surgery, or conventional laparoscopy (4), is the first choice for emergencies as well as for elective procedures, such as cholecystectomies or ectopic pregnancies (1, 5, 6). The known advantages of laparoscopy are a shorter recovery time and hospital stay and less postoperative pain, which favor its use (7). However, laparoscopy requires a producing pneumoperitoneum using CO2 to create the working space in the abdominal cavity (8). Nonetheless, despite the advantages mentioned above, conventional laparoscopy presents some complications or difficulties, such as the need for general anesthesia or hemodynamic and acidobasic alterations caused by pneumoperitoneum, requiring constant monitoring and increasing the cost of surgery (8, 9). This has a great impact in low-income countries, where many patients opt for open surgery or no surgery at all (9).

Given the possible complications of conventional laparoscopy due to pneumoperitoneum, the first publications aimed at solving this problem appeared in the 80s and the 90s. Gasless laparoscopy involves the creation of an intra-abdominal working space (10, 11), by traction of the abdominal wall and sometimes in combination with a low-pressure pneumoperitoneum (10, 12). The first gasless laparoscopic cholecystectomy was performed by Erich Mühe in 1985, after which different methods and devices emerged to achieve this approach (13).

Gasless laparoscopy may be a surgical alternative in countries with fewer economic resources (14), due to its cost savings (9, 15, 16). Several studies included in a systematic review have shown that gasless surgery has similar results to conventional laparoscopy in general surgery and gynecology (17). By not requiring a pneumoperitoneum, there is greater hemodynamic stability, being an option for patients at high cardiovascular risk (16, 18) and may even be considered in patients with an unfavorable American Society of Anesthesiologists classification (19). When comparing the use of conventional laparoscopy with gasless laparoscopy in gynecological pathologies, better operating and bleeding times were reported with the latter approach (20). Therefore, despite the limitations of gasless laparoscopy, this procedure could be a more comfortable alternative with similar results to conventional laparoscopy.

The limitations of gasless laparoscopy described in the literature include low methodological quality and small sample size, limiting the certainty of its results in clinical surgical management (14, 17, 20). Among other important points that are not mentioned in these studies are the severity of the complications identified, the surgeon's experience, or patient comorbidities, precluding correct interpretation of the results and research trends in this field (9). In addition, gasless laparoscopy is expected to have the same versatility as conventional laparoscopy, being a promising line of research (19). Greater dissemination and knowledge of this approach could result in expanding topics of research interest in surgical procedures used in other regions of the world, such as India (7). A bibliometric study, which seeks to analyze trends in article, author and journal output, using qualitative and quantitative indicators, would provide a basis for the main topics addressed, as well as the countries or authors with the highest output in gasless laparoscopy, or how much progress has been made in recent years in this field. However, no such study has been carried out to date (21). For this reason, the present study aimed to establish the bibliometric aspects of the global scientific production of original publications on gasless laparoscopy in the Web of Science Core Collection (WoSCC) platform until 2023.

## 2 Materials and methods

### 2.1 Type of study and source of data

A bibliometric study of original articles on gasless laparoscopic surgery was developed on the WoSCC platform (Science Citation Index Expanded^TM^, Social Sciences Citation Index®, Arts & Humanities Citation Index®, Emerging Sources Citation Index), to develop a performance analysis and bibliometric mapping of the data obtained. WoSCC was used because it has records of articles from the most impactful journals globally and is composed of up to ten citation indexes. WoS coverage has expanded enormously over the years, reaching around 34,000 journals to date (22). WoSCC has also been widely used in bibliometric studies, proving to be a selective, structured and balanced platform with comprehensive citation links and enhanced metadata supporting a wide range of informational purposes (21, 23).

### 2.2 Search strategy

The search and identification of publication records on gasless laparoscopy was performed on January 23, 2024, using the following terms and specifications: TS = (gasless OR “without gas” OR isobaric) AND (laparoscop* OR celioscop* OR peritoneoscop* OR laparoendoscop* OR laparo-endoscop* OR “endoscopic surgery”) and Article (Document Types).

The initial search strategy was developed by three of the authors (A. G. B., P. R. J. and A. H. V.) and then reviewed by a physician specialized in pediatric surgery (R. D. R.), for subsequent approval by all the investigators.

### 2.3 Eligible criteria and data acquisition

A review of the records independently identified in Rayyan was performed by two investigators (A. G. B. and P. R. J.) to assess compliance with each of the study inclusion criteria. These criteria were: original articles addressing the topic of gasless laparoscopy, published up to 2023, articles published in English and Spanish, articles including any research design, original articles that apply to live human models, original articles on abdominopelvic surgery, and original articles on abdominal-pelvic surgery (24). Using the Accession Number of the records obtained in WoSCC, a search was performed on January 28, 2024, to retrieve their metadata and export this information to a Plain Text file. This file was imported into the Notepad program to homogenize the fields of authors (AU), affiliations (C3), and Keywords Plus® to finally obtain a text file that was analyzed.

### 2.4 Statistical data analysis and display

Bibliometric indicators were obtained through the use of the Bibliometrix package in the R programming language (25). VOSviewer 1.6.20 (Leiden University, Leiden, The Netherlands) was also used (26), for the construction of author “networks”, institutional affiliations and Keywords Plus®.

The absolute number of articles, journals, citations, annual number of publications, the ten most cited articles, the journals with the highest number of publications, and the co-authorship networks based on authors, institutional affiliations and Keywords Plus® co-occurrence were presented. The analysis of the networks was developed by means of the full counting method, normalization method by association, node repulsion and node attraction with VOSviewer default values, cluster resolution at 1.00, minimum cluster size at 1, weight according to the number of documents, and term temporality networks based on the average annual publication following the methodology applied in a previous study and the VOSviewer manual (27, 28). For the Keywords Plus® (ID) cooccurrence network, a threshold was applied for cooccurrence in titles and abstracts with at least one mention.

### 2.5 Ethical considerations

The execution of the study did not require the approval of an ethics committee because it was a study using published articles.

## 3 Results

**Figure 1:**
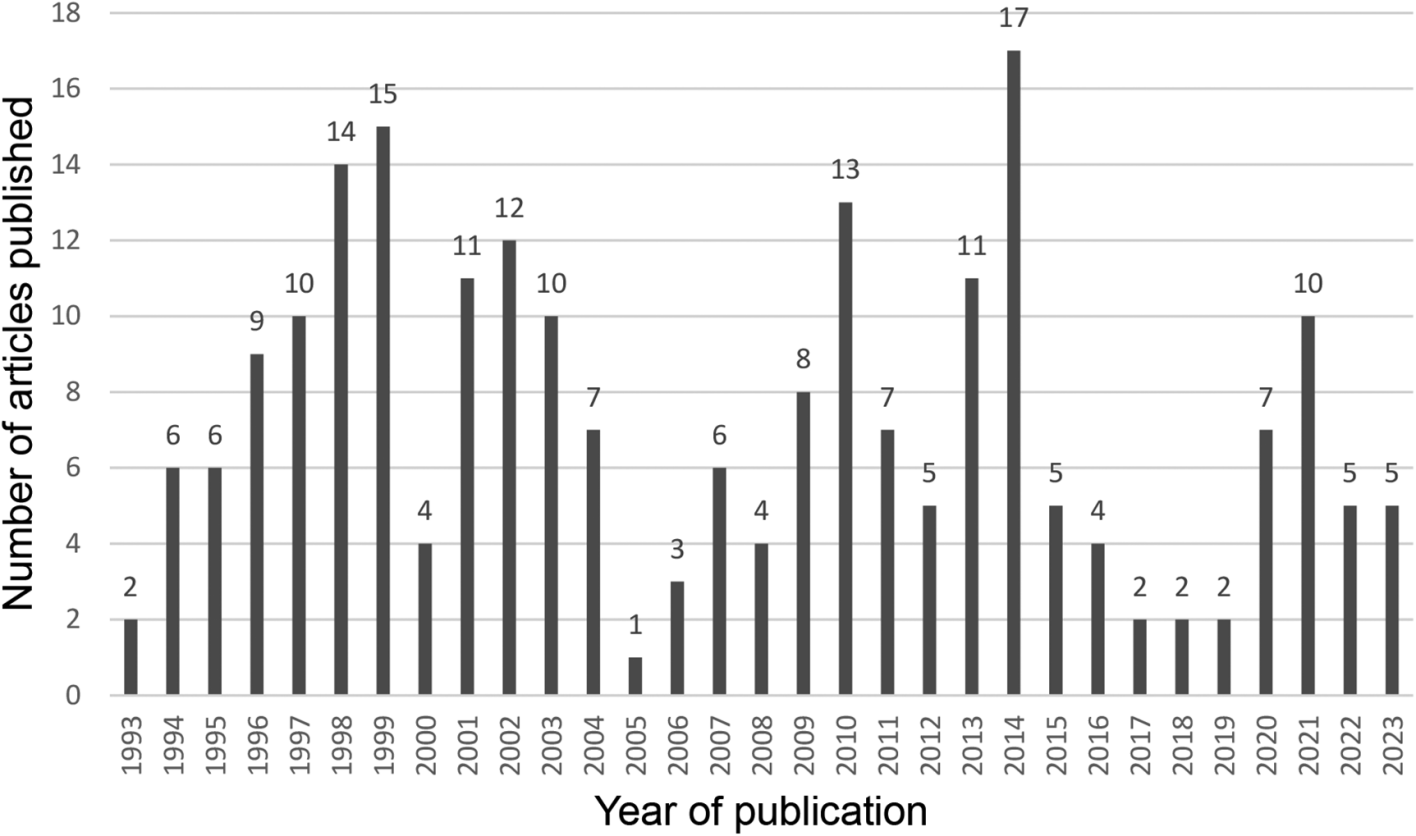
Publication trends of gasless laparoscopy articles in WoSCC 1993–2023.

A search of the WoSCC database yielded a total of 441 articles on gasless laparoscopy up to December 2023. After a review of the titles and abstracts in Rayyan, the sample was reduced to 223 articles, which were included for further analysis. The time period covered was between the years 1993 and 2023, with the years 2014 and 1999 having the highest number of articles published with 17 and 15, respectively (Figure 1). On average, each article has been cited 12.8 times.

### 3.1 Most productive authors

A total of 846 authors were found to be involved in the production of articles on gasless laparoscopy.

Table 1 shows the 10 most productive authors with their corresponding country and the institution with which they are affiliated. It can be seen that Nakamura H., affiliated with “Gifu Prefectural Tajimi Hospital”, stands out as the author with the highest production on gasless laparoscopy with a total of 15 articles between 2007 and 2020. He is followed by Takeda A and Imoto S, with 13 and 10 articles, respectively, both affiliated with the same institution. Of The 10 authors with the highest production, had Japan, Taiwan and Germany as the corresponding countries. Paolucci V. and Gutt CN. presented the greatest production on gasless laparoscopy between 1994 and 1998, being among the authors with greater representation in these years, unlike the others who showed greater production at the beginning of the 21st century.

**Table 1:** Top ten authors with the highest production of articles on gasless laparoscopy in the WoSCC (*N* = 223).

Rank	Author's name	Articles published	% of total publications	Country^a^	Affiliation^a^	H index^a^
1	Nakamura H	15	6.73	Japan	Gifu Prefectural Tajimi Hospital	17
2	Takeda A	13	5.83	Japan	Gifu Prefectural Tajimi Hospital	18
3	Imoto S	10	4.48	Japan	Gifu Prefectural Tajimi Hospital	13
4	Lin MT	10	4.48	Taiwan	National Taiwan University Hospital	43
5	Paolucci V	9	4.04	Germany	Ketteler-Krankenhaus	19
6	Gutt CN	8	3.59	Germany	Klinikum Memmingen	29
7	Yang CY	8	3.59	Taiwan	National Taiwan University Hospital	36
8	Kihara K	7	3.14	Japan	Tokyo Medical and Dental University	41
9	Mori M	7	3.14	Japan	Graduate School of Medicine	83
10	Wang MY	7	3.14	Taiwan	National Taiwan University Hospital	24

NA, not available.

ª Collected from WoSCC.

### 3.2 Articles and journals

Table 2 lists the 10 most cited articles on gasless laparoscopy, being “Hemodynamic and pulmonary changes during open, carbon dioxide pneumoperitoneum and abdominal wall-lifting cholecystectomy. A prospective, randomized study”, the first on the list with 132 citations, published by Galizia G in 2001. The second most cited article is “Gasless laparoscopy and conventional instruments. The next phase of minimally invasive surgery” by Smith R S in 1993 with a total of 99 citations.

**Table 2:** Top ten most cited articles on gasless laparoscopy in WoSCC 1993–2023.

Rank	Title	Authors	Year of publication	Journal	Total citations	Citations per year	NTC
1	Hemodynamic and pulmonary changes during open, carbon dioxide pneumoperitoneum, and abdominal wall-lifting cholecystectomy. A prospective, randomized study	Galizia G et al.	2001	Surgical endoscopy and other interventional techniques	132	5.50	5.17
2	Gasless laparoscopy and conventional instruments. The next phase of minimally invasive surgery	Smith RS et al.	1993	Archives of surgery	99	3.09	1.60
3	Splanchnic and renal deterioration during and after laparoscopic cholecystectomy: a comparison of the carbon dioxide pneumoperitoneum and the abdominal wall lift method	Koivusalo AM et al.	1997	Anesthesia and analgesia	76	2.71	3.30
4	A comparison of gasless mechanical and conventional carbon dioxide pneumoperitoneum methods for laparoscopic cholecystectomy	Koivusalo AM et al.	1998	Anesthesia and analgesia	65	2.41	4.08
5	Gasless laparoscopic cholecystectomy: comparison of postoperative recovery with conventional technique	Koivusalo AM et al.	1996	British Journal of Anaesthesia	63	2.17	3.61
6	Randomized clinical trial of the effect of pneumoperitoneum on cardiac function and haemodynamics during laparoscopic cholecystectomy	Larsen JF et al.	2004	British Journal of Surgery	61	2.90	3.34
7	Cardiorespiratory effects of laparoscopy with and without gas insufflation	McDermott JP et al.	1995	Archives of surgery	58	1.93	2.81
8	Gasless laparoscopic ovarian cystectomy during pregnancy: comparison with laparotomy	Akira S et al.	1999	American journal of obstetrics and gynecology	55	2.12	2.21
9	Randomized comparison between low-pressure laparoscopic cholecystectomy and gasless laparoscopic cholecystectomy	Vezakis A et al.	1999	Surgical endoscopy and other interventional techniques	52	2.00	2.09
10	Changes in urinary output during laparoscopic adrenalectomy	Nishio S et al.	1999	BJU International	45	1.73	1.81

NTC: total number of citations/average n° of citations of all documents published in the same years.

A total of 100 sources of information were found. Table 3 shows the 10 journals with the highest number of publications on gasless laparoscopy. The first place is held by Surgical Endoscopy-Ultrasound and Interventional Techniques with 20 articles followed by Surgical Endoscopy and Other Interventional Techniques and the Journal of Laparoendoscopic & Advanced Surgical Techniques, with 14 and 10 articles, respectively.

**Table 3:** Top ten journals with publications on gasless laparoscopy (*N* = 223) WoSCC 1993–2023.

Rank	Source title	Articles published	% of articles	Quartile categoryª
1	Surgical endoscopy-ultrasound and interventional techniques^b^	20	8.97	NA
2	Surgical endoscopy and other interventional techniques^b^	14	6.28	Q1
3	Journal of laparoendoscopic & advanced surgical techniques	10	4.48	Q4
4	Hepato-gastroenterology	9	4.04	Q4
5	Journal of minimally invasive gynecology^c^	9	4.04	Q1
6	European journal of obstetrics & gynecology and reproductive biology	8	3.59	Q3
7	Journal of the American Association of gynecologyc laparoscopists	6	2.69	NA
8	Surgical laparoscopy endoscopy & percutaneous techniques^c^	6	2.69	Q4
9	International journal of urology	5	2.24	Q3
10	JSLS-Journal of the Society of laparoendoscopic surgeons	5	2.24	NA

NA, not available.

ª Collected from WoSCC.

^b^ Both journals correspond to the same one, the current name being surgical endoscopy and other interventional techniques.

^c^ Both journals correspond to the same one, the current name being journal of minimally invasive gynecology.

### 3.3 Most productive countries

Table 4 shows the top 10 correspondent countries of the authors in terms of production on gasless laparoscopy, of which 5 belong to Asia, 4 to Europe and 1 to the United States. Japan is the country with the highest production with 64 articles, followed by China with 46, and Italy and the United States with 18 articles each.

**Table 4:** The top ten corresponding author countries with the most articles published on gasless laparoscopy in the WoSCC 1993–2023 (*N* = 223).

Rank	Country	Articles	% of articles	SCP	MCP
1	Japan	64	28.70	64	0
2	China	46	20.63	45	1
3	Italy	18	8.07	18	0
4	United States	18	8.07	15	3
5	Germany	12	5.38	12	0
6	Korea	10	4.48	9	1
7	Turkey	6	2.69	6	0
8	Denmark	5	2.24	5	0
9	Thailand	4	1.79	4	0
10	United Kingdom	4	1.79	0	4

SCP, single country publications; MCP, multiple country publications.

In terms of the number of citations per article, Table 5 shows that Japan remains in the lead with 741 in total with an average of 11.58 citations per article, followed by Italy with 371 and 20.61, respectively. However, countries such as Finland, Denmark and Greece published articles with higher impact, presenting a higher average number of citations of 70.50, 31.00 and 52.00, respectively.

**Table 5:** The top ten countries with the most cited articles published on gasless laparoscopy in the WoSCC 1993–2023.

Rank	Country	Total citations	Average article citations
1	Japan	741	11.58
2	Italy	371	20.61
3	United States	358	19.89
4	China	350	7.61
5	Denmark	155	31.00
6	Finland	141	70.50
7	Germany	77	6.42
8	Turkey	68	11.33
9	Sweden	55	27.50
10	Greece	52	52.00

### 3.4 Most used key terms

**Figure 2:**
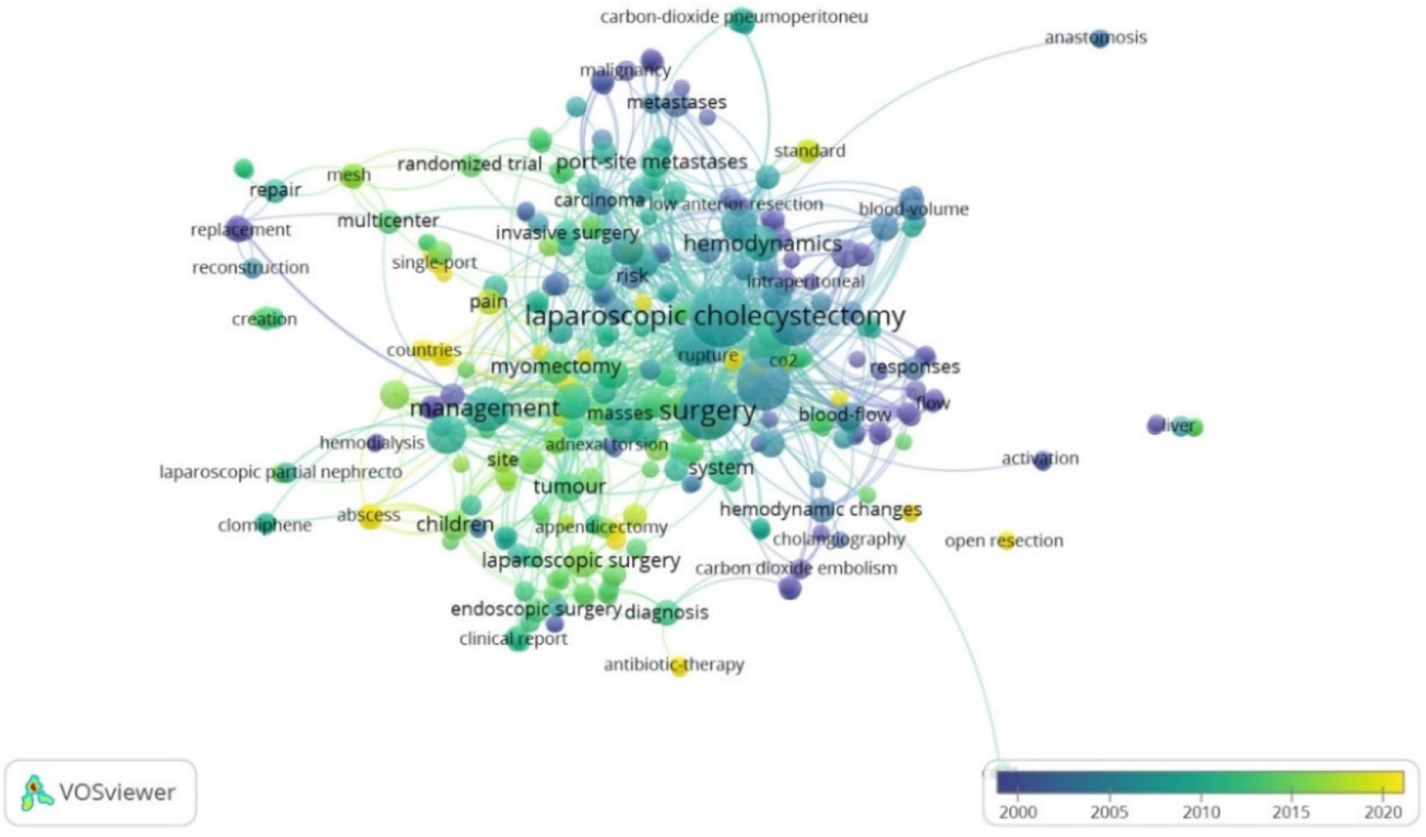
Network of the most frequently used key terms by year.

**Figure 3:**
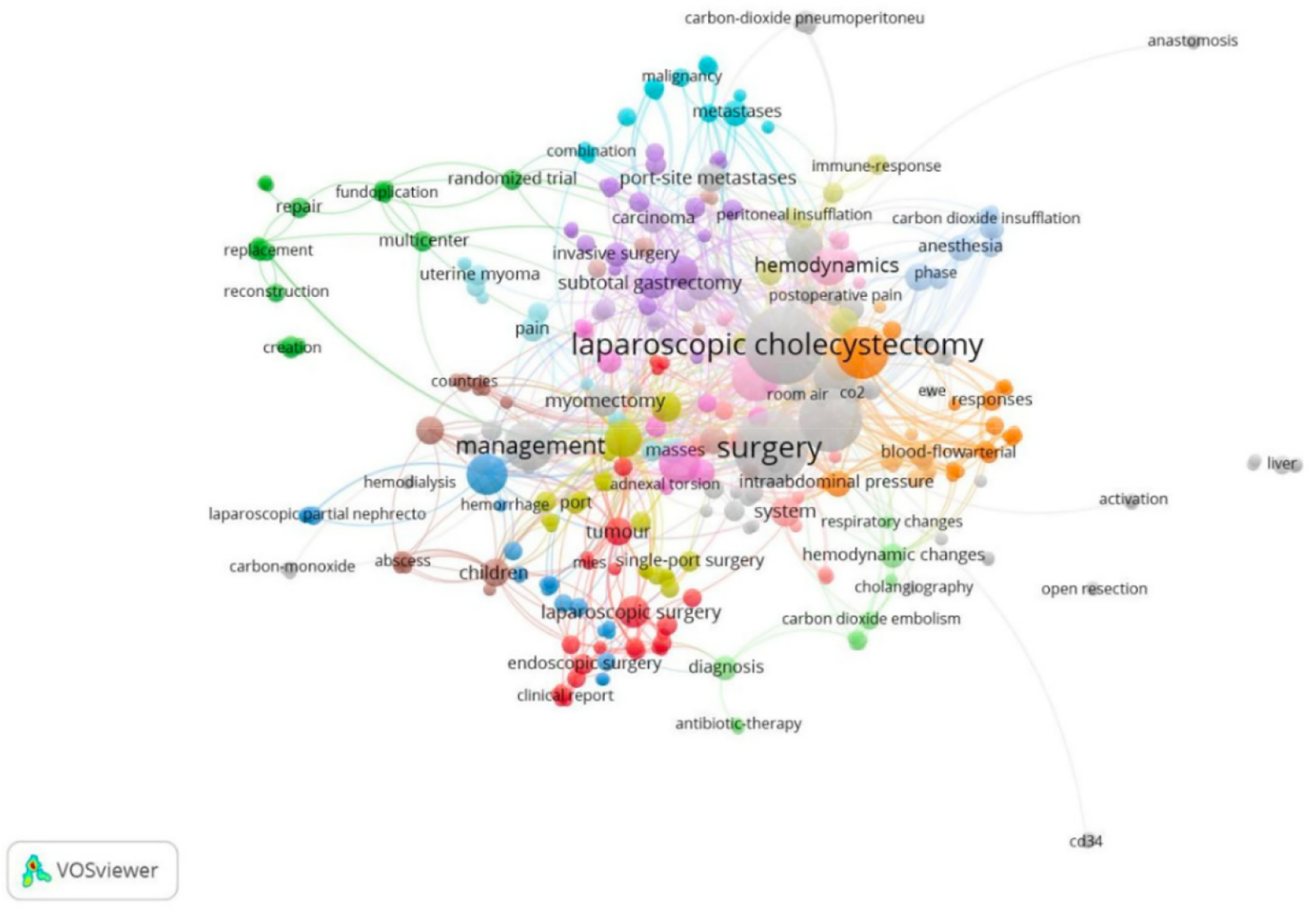
Network of terms grouped by co-occurrence in WoSCC.

In terms of keywords, 385 terms were identified. Figure 2 shows the network of the most frequently used key terms by year. We found that the most used terms were “laparoscopic cholecystectomy”, “management” and “surgery”, especially between 2005 and 2010. The terms most present at the beginning of the century were “malignancy”, “metastases”, “carbon dioxide embolism” and “blood-flow”; as opposed to those most used in recent years such as “single-port”, “children”, “pain” or “antibiotic-therapy”. Likewise, Figure 3 presents the network of terms grouped by co-occurrence, showing that terms such as “carcinoma”, “port-site metastases” or “invasive surgery” appear together. Likewise, “respiratory changes”, “hemodynamic changes” and “carbon dioxide embolism” also tend to appear together.

### 3.5 Most productive institutions

**Figure 4:**
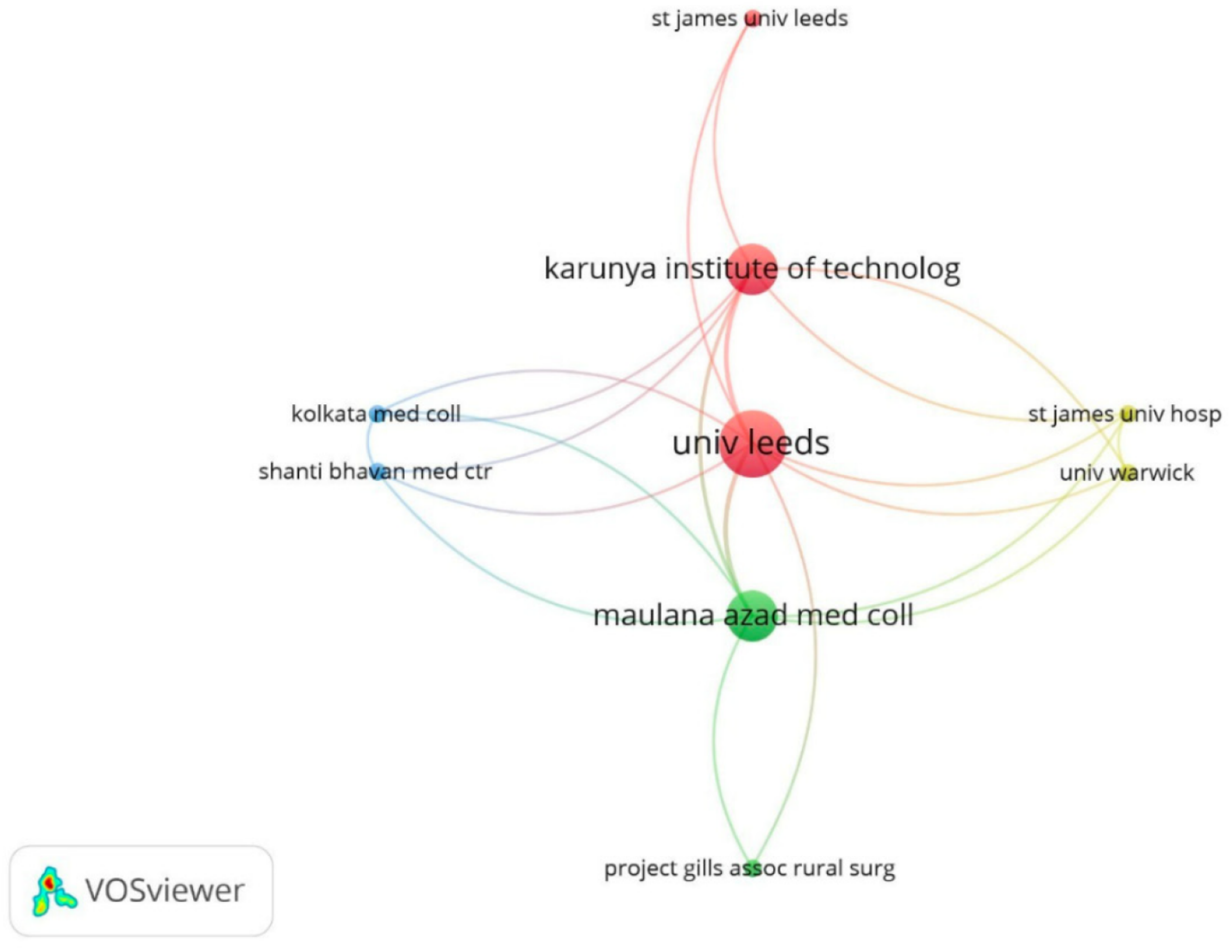
Network of institutional affiliations with the highest production on gasless laparoscopy in WoSCC.

Regarding institutions, Figure 4 shows the network of affiliations producing literature on gasless laparoscopy. The most relevant institutions are the University of Leeds (29) in England and Maulana Azad Medical College together with the Karunya Institute of Technology and Sciences in India. These three institutions show multiple collaborations with other organizations and with each other.

## 4 Discussion

Despite the demonstrated benefits of gasless laparoscopy, the production of articles on this subject has stagnated over the years. It is possible that the improvement in surgical or anesthesiological management of situations that might contraindicate pneumoperitoneum has favored conventional laparoscopy to remain as the intervention of choice.

We found that 1999 and 2014 were the years in which the largest number of articles were published, showing a sustained trend in the number of articles published throughout the decades. Likewise, data corresponding to authors with the highest production were identified, as well as the countries and terms that were most relevant throughout all the years of production.

Nakamura H is the author with the most publications, with all the research having been conducted at the “Gifu Prefectural Tajimi Hospital” in Japan. With a total of 15 articles between 2007 and 2020, this author is among those with the greatest range of activity over time. The topics addressed by Nakamura remain consistent, dealing mostly with the laparoscopic management of adnexal tumors through a single incision, as shown in the publication of a study in 2011 including a series of 100 cases. The latest publications by Nakamura consist of case reports involving the treatment of pregnant women and even the successful management of ectopic pregnancies (30).

The most cited article is “Hemodynamic and pulmonary changes during open, carbon dioxide pneumoperitoneum and abdominal wall-lifting cholecystectomy. A prospective, randomized study” published by Galizia G in 2001 with a total of 132 citations. This study was published in the journal Surgical Endoscopy and Other Interventional Techniques which has the second highest number of publications on gasless laparoscopy, with 14 in total. The study by Galizia consisted in the measurement of various cardiovascular parameters in three study groups, maintaining the same conditions in all the groups and altering only the surgical approach. Thus, the results of this study were completely objective and with few biases, making this study a reference and a good basis for further research (31).

Regarding the most productive journals, it was of note that they were focused on specialized aspects of surgery rather than general aspects, which could have greater diffusion. As expected, surgical journals were more oriented towards conventional laparoscopy since gasless laparoscopy is a less known or used technique and may not be of interest to a journal with broader vision.

Japan leads the scientific production with a total 64 articles on gasless laparoscopy which, according to the corresponding authors, focus mainly on the evaluation of this technique in gynecological pathologies. In addition, in the period from 2014 to 2020 the number of laparoscopic hysterectomies increased considerably from 16,016 to 27,755, observing a trend towards the use of minimally invasive interventions which corresponded to more than 50% of the hysterectomies performed since 2019 (32). This makes this country an important niche for research in this field. On the other hand, according to Scimago Journal and Country Rank, Japan ranks seventh in scientific production until 2020 (33). China, follows the same ranking, occupying second place in scientific production on gasless laparoscopy. In contrast to these results, no Latin American country is included among the top ten authors in terms of number of publications. This may be due to the lesser research funding which mainly comes from the government in this region, and to the fact that socioeconomic conditions do not favor investment in research (34). Despite this, given the inequality in the region, gasless laparoscopy should be considered a viable option (35). However, a possible lack of knowledge of this technique, lack of surgeons who have the necessary expertise to train other physicians or the fact of centralizing surgical interventions, may be factors that prevent the investigation and even the implementation of this technique in our setting.

On the other hand, the publications on gasless laparoscopy with the highest impact belong to countries such as Finland, Greece or Denmark, which is probably due to the methodology used or the objectives set. The Finnish author Koivusalo AM stands out with three of the five most cited articles. These studies present a reliable methodology since they apply a strict control of both the pre-surgical interventions and the target parameters during and after surgery (36–38). This author presented relevant results related to the length of the procedure, demonstrating the benefits of gasless laparoscopy over conventional laparoscopy in the involvement of various organs.

With respect to the keywords found, initially terms such as “malignancy” or “metastases” predominated, showing the interest in the earlier years in the risk of dissemination due to the use of conventional laparoscopy in oncologic pathologies caused by pneumoperitoneum (39). Several publications at that time reported a risk of dissemination and recurrence of oncologic pathologies at the trocar insertion sites after laparoscopy (40, 41), which led to interest in the use of gasless laparoscopy in cancer management. However, Mo X et al. conducted a meta-analysis in 2014 refuting an increased risk for recurrence (42). In recent years, the most used terms are “single-port”, “children” or “antibiotic-therapy” showing the new tendency of authors to combine this gasless surgical technique with the use of fewer access points, or to extend its application to children.

We also evaluated the networks of terms that were grouped in clusters according to the line of research or areas of application of gasless laparoscopy. The complications of pneumoperitoneum have been an important area of study in gasless laparoscopy, since terms such as “respiratory changes”, “hemodynamic changes” or “carbon dioxide embolism” have frequently been mentioned, demonstrating that gasless laparoscopy presents an advantage by having minimal alterations in these parameters (43). The network of terms “subtotal gastrectomy”, “invasive surgery”, “carcinoma” or “port-site metastases”, suggests research on the use of this technique in surgical-oncological management, in which it has been successfully used in different gastrointestinal pathologies (44). Likewise, its relationship with terms such as “uterine myoma” and “myomectomy” indicates its application in gyneco-oncologic pathologies, in which the aim is to provide more precise information to help choose the most appropriate surgical approach according to the characteristics of the patient to be treated (45).

As for affiliations, again there was an absence of Latin American institutions, reaffirming the lack of research in this field in the region. Institutions belonging to India or England stand out for great mutual cooperation, all being educational institutions. However, India is not among the most productive. This can be explained by the fact that probably, the corresponding authors of the publications were from another institution and the rest were omitted for the analysis. Project GILLS is a project carried out by surgeons in rural India that aims to increase the number and variety of surgical interventions in the area (46). Different organizations can collaborate with the project either financially or with material or human resources, and among these collaborators there is the University of Leeds, thus explaining its position as the most productive institution.

Gasless laparoscopy has demonstrated benefits in reducing hospital costs and hospitalization days (15, 17). Specifically, gasless laparoscopy for appendectomy has shown similar results to conventional laparoscopy in terms of operative time, surgical complications, and hospital stay, with the added advantage of lower hospital costs (15). Although it presents a higher conversion rate compared to conventional laparoscopy in gynecological surgeries, gasless laparoscopy still results in shorter hospitalization periods compared to conventional laparoscopic abdominal and gynecological surgeries (17). Moreover, due to its lower associated costs, the gasless laparoscopic technique can be implemented in low-income countries, as has been successfully done in India (7).

Currently, energetic vessel sealing devices, such as the harmonic scalpel, are used in laparoscopic procedures. Their use in laparoscopic cholecystectomy, compared to other hemostasis management devices, has shown benefits including shorter hospital stays, reduced operative time, fewer perioperative complications, and less postoperative pain (47). A systematic review also indicates similar advantages of these new energy devices over monopolar or bipolar devices in gynecologic laparoscopy (48). While these new energy devices (harmonic scalpel, LigaSure, EnSeal) may incur higher costs than monopolar or bipolar devices, the cost savings associated with gasless laparoscopy could offset these expenses. Therefore, the use of energetic devices in gasless laparoscopy warrants evaluation in future prospective studies.

Regarding the limitations of our study, the use of WoSCC as the only database means that studies published in other databases, such as Scopus or MEDLINE, may have been omitted. It is known that different databases have a varied coverage of journals according to geographical areas. Scopus and WoSCC have been shown to under-record journals from Africa and South America compared to other regions of the world (49). Another limitation is the exclusion of articles applied to animal or cadaveric models, which could create a bias in our analysis as it is possible that important information, such as new methods or applications of this surgical technique, could be omitted. However, WoS offers a large amount of data and information about the articles, which increases the quality of the analysis of the journals and has allowed standardization of the data. Furthermore, approximately 99.11% of the journals indexed in WoSCC are also indexed in Scopus, which does not cause significant differences (50). Additionally, documents in languages other than English may have been ignored since only English documents were included, potentially overlooking relevant studies published in other languages. In the future research, literature related to gasless laparoscopy should be collected from multiple bibliographic databases to provide a more comprehensive overview of scientific production in this field.

In conclusion, the results of the present study show a low and oscillating production of scientific output on gasless laparoscopy. Initially the focus of the studies on this technique was on the complications of pneumoperitoneum and the feasibility of the technique. Later research is aimed at new applications of gasless laparoscopy, such as single incision laparoscopy or its application in children. The most productive authors are located in Japan, the country with the highest number of publications according to the corresponding author. To date, this is the first study analyzing the scientific production on gasless laparoscopy, showing the current state of research in this field and laying the groundwork for possible future publications.

## Data Availability

The original contributions presented in the study are included in the article/Supplementary Material, further inquiries can be directed to the corresponding author.
